# Traditional medicine practices among community members with diabetes mellitus in Northern Tanzania: an ethnomedical survey

**DOI:** 10.1186/s12906-016-1262-2

**Published:** 2016-08-11

**Authors:** Joseph Lunyera, Daphne Wang, Venance Maro, Francis Karia, David Boyd, Justin Omolo, Uptal D. Patel, John W. Stanifer, for the Comprehensive Kidney Disease Assessment For Risk factors, epidemiology, Knowledge, and Attitudes (CKD AFRiKA) Study

**Affiliations:** 1 Duke Global Health Institute, Duke University, 310 Trent Drive, Durham, NC 27705 USA; 2 Kilimanjaro Christian Medical University College, Moshi, Tanzania; 3 National Institute for Medical Research, Dar es Salaam, Tanzania; 4 Departments of Medicine and Pediatrics, Duke Clinical Research Institute, Durham, NC USA; 5 Division of Nephrology, Department of Medicine, Duke University School of Medicine, Durham, NC USA; 6 Duke Clinical Research Institute, Duke University, Durham, NC USA

**Keywords:** Biomedicine, Traditional medicine, Sub-Saharan Africa, Low- and middle-income countries, Non-communicable diseases

## Abstract

**Background:**

Diabetes is a growing burden in sub-Saharan Africa where traditional medicines (TMs) remain a primary form of healthcare in many settings. In Tanzania, TMs are frequently used to treat non-communicable diseases, yet little is known about TM practices for non-communicable diseases like diabetes.

**Methods:**

Between December 2013 and June 2014, we assessed TM practices, including types, frequencies, reasons, and modes, among randomly selected community members. To further characterize TMs relevant for the local treatment of diabetes, we also conducted focus groups and semi-structured interviews with key informants.

**Results:**

We enrolled 481 adults of whom 45 (9.4 %) had diabetes. The prevalence of TM use among individuals with diabetes was 77.1 % (95 % CI 58.5–89.0 %), and the prevalence of using TMs and biomedicines concurrently was 37.6 % (95 % CI 20.5–58.4 %). Many were using TMs specifically to treat diabetes (40.3 %; 95 % CI 20.5–63.9), and individuals with diabetes reported seeking healthcare from traditional healers, elders, family, friends, and herbal vendors. We identified several plant-based TMs used toward diabetes care: *Moringa oleifera*, *Cymbopogon citrullus*, *Hagenia abyssinica, Aloe vera, Clausena anisata, Cajanus cajan, Artimisia afra*, and *Persea americana*.

**Conclusions:**

TMs were commonly used for diabetes care in northern Tanzania. Individuals with diabetes sought healthcare advice from many sources, and several individuals used TMs and biomedicines together. The TMs commonly used by individuals with diabetes in northern Tanzania have a wide range of effects, and understanding them will more effectively shape biomedical practitices and public health policies that are patient-centered and sensitive to TM preferences.

**Electronic supplementary material:**

The online version of this article (doi:10.1186/s12906-016-1262-2) contains supplementary material, which is available to authorized users.

## Background

The prevalence of diabetes mellitus (DM) is rapidly growing globally with a disproportionate burden falling on low- and middle-income regions [1]. In sub-Saharan Africa, DM prevalence is now as high as 18 % in some countries and current estimates portend that the prevalence of DM in the region could nearly double by the year 2045, which means that more than 40 million people would be living with diabetes in sub-Saharan Africa [2]. Despite this, access to biomedical healthcare remains limited for most populations in the region [3, 4]. Health systems and healthcare clinics are currently under-prepared to adequately meet the demands of caring for non-communicable diseases (NCDs) such as DM [5, 6]. In this setting, traditional medicines (TMs), which are defined by the World Health Organization (WHO) as “the sum total of the knowledge, skills, and practices based on the theories, beliefs, and experiences indigenous to different cultures, whether explicable or not, used in the maintenance of health as well as in the prevention, diagnosis, improvement or treatment of physical and mental illness [7],” are critical in meeting the healthcare needs for much of the population [8]. The WHO World Health Assembly and regional committees, including the WHO African regional committee and African heads of states, have adopted resolutions which formally recognized TMs as pivotal in health service delivery. They also urged governments to integrate TMs into their health system based on innovation and research [7, 9–11]. However, in the context of NCDs, for the most part these resolutions remain unmet due to limited understanding of population-based TM practices [12, 13].

In Tanzania, TM use is frequent with more than half of all adults regularly using TM [14]. They are used for treating a variety of conditions, including NCDs [12, 15]. However, little is known about the types, modes, frequencies, reasons for use, and pharmacology of TMs used to manage NCDs especially as they pertain to DM [12–16]. Considering the burden of DM, characterizing TM practices among community members with and at risk for DM will be important in formulating optimal disease management programs and public health efforts in the region [13].

As part of the Comprehensive Kidney Disease Assessment for Risk Factors, epidemiology, Knowledge, and Attitudes (CKD AFRiKA) study in Northern Tanzania, we conducted assessments among community-based adults using focus-group discussions, in-depth interviews, and structured surveys. We sought to characterize the use of TMs among a community-based population in order to help shape public health efforts and biomedical practices addressing DM in ways that are sensitive to local TM practices. Specifically, we described TM practices among community members with DM including the types, frequencies, modes, and pharmacological descriptions of those relevant to DM care.

## Methods

### Study setting

The CKD AFRiKA Study was conducted in December 2013-June 2014 and March-June 2015 in the Kilimanjaro Region of Tanzania. The region has an adult population of 900,000 for which KCMC hospital serves as the tertiary referral hospital. Patients pay a standard fee scaled to their income for their clinical care at the hospital and the hospital-based clinics [17]. The majority of the population lives in rural settings (65 %). The unemployment rate is 19 %, and the majority of adults have a primary education or less (77 %). The largest ethnic group is the Chagga tribe followed by the Pare, Sambaa, and Maasai tribes. Swahili is the major language, and all participants in our study spoke it as their first language [18].

### Quantitative data collection

We developed a structured survey instrument designed to test different factors related to TM practices among community members (Additional file 1). The development of the survey instrument has been previously described [14, 19]. In brief, the instrument was drafted in English by local and non-local experts from the fields of medicine, epidemiology, sociology, anthropology, and public health. It was independently translated into Swahili by two native speakers with joint review of each version. We piloted it through several qualitative sessions in an iterative process in order to ensure the content validity of the survey instrument. The final version of the survey instrument included nine items including both open-ended questions related to types of TMs used by community members as well as close-ended questions related to frequency of use, reasons for use, modes of use, modes of access, and conditions treated by TMs.

Using two local surveyors, we verbally administered the survey to adult community members from the Moshi Urban and Moshi Rural districts of the Kilimanjaro Region. The overall sample size was based on the requirements of the CKD AFRiKA study, which was designed to estimate the community prevalence of chronic kidney disease with a precision of 5 % when accounting for the cluster-design effect. Using a random-number generator, we selected thirty seven sampling areas from twenty-nine neighborhoods, stratified by urban and rural. We based the random neighborhood selection on probability proportional to size using the 2012 Tanzanian National Census [18]. Within each neighborhood the sampling area was determined using geographic points randomly generated using Arc Global Information Systems (ArcGIS), v10.2.2 (Environmental Systems Research Institute, Redlands, CA), and households were then randomly chosen based on coin-flip and die-rolling techniques [20]. All adults living in the selected households were recruited, and all Tanzanian citizens over the age of 18 were eligible for inclusion. To reduce non-response rates, we attempted a minimum of two additional visits on subsequent days and weekends, and using mobile phone numbers, we located eligible participants through multiple phone calls.

### Qualitative data collection

As part of the CKD AFRiKA Study, we conducted focus group discussions (FGDs) and in-depth interviews in a central, easily accessible location. These sessions have been described previously, but in brief, we conducted five FGDs and 27 in-depth interviews both of which included key informants from the community including well-adults from the general population, chronically-ill adults receiving care at the hospital medicine clinics, adults receiving care from traditional healers, adults purchasing TMs from herbal vendors, traditional healers, herbal vendors, and medical doctors [14].

### Disease definitions

Participants were assessed for DM as part of the CKD AFRiKA Study [20]. Hemoglobin (Hb) A1c was measured from a fingerstick whole blood sample using the Bayer A1c Now+ point-of-care device (Bayer Healthcare LLC; Sunnyvale, CA). DM was considered present if the HbA1c level was greater than 7.0 % (53 mmol/mol) or current known use of anti-hyperglycemic medications for the purpose of treating diabetes. We considered participants to be at increased risk for diabetes if their HbA1c was between 6.0 % (42 mmol/mol) and 6.9 % (52 mmol/mol) in the absence of ongoing treatment with anti-hyperglycemic medicines.

### Data analysis and management

Quantitative data were analyzed using STATA v.13 (STATA Corp., College Station, TX). The median and inter-quartile ranges (IQR) were reported for continuous variables. All *p* values are two-sided at a 0.05 significance level. To compare differences between groups, we used a Chi squared test or Fisher’s Exact test. Prevalence estimates were sample-balanced using age- and gender-weights based on the 2012 urban and rural district-level census data, and they are reported with 95 % confidence intervals (CI) [21]. We used Taylor Series linearization to account for the design effect due to cluster sampling. All data were collected on paper and then electronically entered into and managed using REDCap electronic data capture tools hosted at Duke University. REDCap is a secure, web-based application designed to support data capture for research studies [21]. All data were verified after electronic data entry by an independent reviewer to ensure accuracy.

To identify traditional medicines used for the treatment of DM, two authors (JL and DW) independently reviewed the transcripts of the FGDs and in-depth interviews conducted as part of the CKD AFRiKA Study. All TMs referenced by participants were recorded in the coding index, and analytic memos were created for TMs referenced for treatment of DM. A third author (JO) familiar with local languages, dialects, and customs, then cross-referenced the local vernacular with known botanical and linguistic catalogues in the region and country. Any discrepancies were resolved by joint consensus. All of the qualitative data, including the coding index, were recorded and managed using NViVO v.10.0 (QRS International Pty Ltd, Melbourne, Australia).

## Results

### Demographics of study participants

We enrolled 481 participants in the CKD AFRiKA Study. Forty-five participants (9.4 %) had DM, and 84 (17.5 %) were at increased risk for DM. The household non-response rate was 15.0 %, and the individual non-response rate was 21.0 %. Compared to the regional census data, men (*p* < 0.001) and young adults (18–39 years old; *p* = 0.001) were more likely to be non-responders [18]. The most common reason for non-response was inability to locate or contact individuals.

Adults with DM were mostly female (*n* = 30; 66.7 %), ≥40 years of age (*n* = 40; 88.9 %), urban residents (*n* = 39; 86.7 %), ethnically Chagga (*n* = 26; 57.8 %), Protestant (*n* = 22; 48.9 %), and had a primary school education (*n* = 26; 57.8 %) (Table 1). The median age of adults with DM was 59 (IQR 48–68) years old. Several participants with DM were unaware of having it (*n* = 16; 35.6 %), but many did report a history of hypertension (*n* = 21; 46.7 %). Few reported a history of heart disease, kidney disease, stroke, chronic obstructive pulmonary disease (COPD), or human immunodeficiency virus (HIV). Alcohol use was common (*n* = 28; 62.2 %) but few reported a history of smoking (*n* = 13; 28.9 %).

**Table 1:** Participant characteristics by diabetes and diabetes risk status; CKD-AFRIKA study population, 2014; *N* = 481

Variable	Participants				
	Overall (*n* = 481)	Diabetes Absent (*n* = 436)		Diabetes Present (*n* = 45)	*p*-value*
		Low risk (*n* = 352)	Increased risk (*n* = 84)		
Gender					0.21
Male	123 (26 %)	89 (25 %)	19 (23 %)	15 (33 %)	
Female	358 (74 %)	263 (75 %)	65 (77 %)	30 (67 %)	
Age					<0.01
18–39 years old	172 (36 %)	143 (41 %)	24 (29 %)	5 (11 %)	
40–59 years old	191 (40 %)	135 (38 %)	37 (44 %)	19 (42 %)	
60+ years old	118 (24 %)	74 (21 %)	23 (27 %)	21 (47 %)	
Setting					0.10
Rural	111 (23 %)	84 (24 %)	21 (25 %)	6 (13 %)	
Urban	370 (77 %)	268 (76 %)	63 (75 %)	39 (87 %)	
Ethnicity					0.50
Chagga	288 (60 %)	217 (62 %)	45 (54 %)	26 (58 %)	
Pare	66 (14 %)	43 (12 %)	19 (23 %)	4 (9 %)	
Sambaa	27 (6 %)	22 (6 %)	3 (4 %)	2 (4 %)	
Other^a^	100 (20 %)	70 (20 %)	17 (20 %)	13 (29 %)	
Religion					0.18
Roman Catholic	192 (40 %)	149 (42 %)	31 (37 %)	12 (27 %)	
Protestant	161 (33 %)	117 (33 %)	22 (26 %)	22 (49 %)	
Islam	123 (26 %)	84 (24 %)	28 (33 %)	11 (24 %)	
Hindu	2 (<1 %)	1 (<1 %)	1 (1 %)	0 (0 %)	
Education					0.06
None	31 (6 %)	21 (6 %)	4 (5 %)	6 (13 %)	
Primary	349 (73 %)	260 (74 %)	63 (75 %)	26 (58 %)	
Secondary	74 (15 %)	52 (15 %)	12 (14 %)	10 (22 %)	
Post-Secondary	27 (6 %)	19 (5 %)	5 (6 %)	3 (7 %)	
Occupation					<0.01
Unemployed	74 (15 %)	52 (15 %)	15 (18 %)	7 (16 %)	
Farmer/Wage Earner	199 (41 %)	157 (45 %)	32 (38 %)	10 (22 %)	
Small Business/Vendors	158 (33 %)	121 (34 %)	25 (30 %)	12 (27 %)	
Professional^b^	50 (10 %)	22 (6 %)	12 (14 %)	16 (36 %)	
History of Smoking	117 (24 %)	85 (24 %)	19 (23 %)	13 (29 %)	0.45
History of alcohol intake	318 (66 %)	236 (67 %)	54 (64 %)	28 (62 %)	0.56
Self-Reported Medical History					
Hypertension	134 (28 %)	86 (25 %)	27 (32 %)	21 (47 %)	<0.01
Diabetes	61 (13 %)	23 (7 %)	9 (11 %)	29 (64 %)	<0.01
Heart Disease^c^	18 (4 %)	10 (3 %)	3 (4 %)	5 (11 %)	0.01
HIV	21 (4 %)	21 (6 %)	0 (0 %)	0 (0 %)	0.24
Stroke	8 (2 %)	6 (2 %)	0 (0 %)	2 (4 %)	0.17
COPD	8 (2 %)	6 (2 %)	1 (1 %)	1 (2 %)	0.55
Kidney Disease	14 (3 %)	7 (2 %)	3 (4 %)	4 (9 %)	0.03

*COPD* Chronic obstructive pulmonary disease

*P-value comparing differences by diabetes status (present or absent)

^a^Other ethnicities includes Maasai, Luguru, Kilindi, Kurya, Mziguwa, Mnyisanzu, Rangi, Jita, Nyambo, and Kaguru

^b^Professional included any salaried position (e.g. nurse, teacher, government employee, etc.) and retired persons

^c^Heart disease included coronary disease, heart failure, or structural diseases

Among those at increased risk for DM (*n* = 84), most were female (*n* = 65; 77.4 %), ethnically Chagga (*n* = 45; 54 %), urban residents (*n* = 63; 75.0 %), and had a primary school education (*n* = 63; 75.0 %). Unlike those with diabetes, they were younger (median age = 42 years; IQR 33–56)(*p* < 0.01) and were more likely to be occupied as farmers or wage earners (*n* = 32; 38.1 %)(*p* < 0.01).

### Epidemiology of traditional medicine use

The prevalence of adults with DM reporting any TM use was 77.1 % (95 % CI 58.5–89.0 %) (Table 2). Most reported using TM 1–5 times per year (45.3 %; 95 % CI 27.8–64.0 %), but many also reported use of more than 10 times per year (26.7 %; 95 % CI 10.2–53.8 %). Fewer than half of adults with DM reported ongoing use of any biomedicine (42.2 %; 95 % CI 24.3–62.4 %), and among these, nearly three-quarters (*n* = 13; 72.2 %) were also using TMs. As such, among all participants with DM, the prevalence of concurrent use of TM and biomedicine was 37.6 % (95 % CI 20.5–58.4 %), which did not vary significantly by age, gender, occupation, or education (all *p*-values > 0.05).

**Table 2:** Epidemiology and characteristics of traditional medicine use stratified by diabetes and diabetes risk status; CKD-AFRIKA

	Diabetes Absent; *n* = 436 (95 % CI)		With Diabetes; *n* = 45 (%, 95 % CI)
	Low risk (*n* = 352)	Increased risk (*n* = 84)	
Prevalence			
…of TM Use	60.3 % (48.9–70.7)	56.7 % (43.4–69.2)	77.1 % (58.5–89.0)
…of concurrent TM and Biomedicine Use	4.9 % (2.7–8.9)	2.6 % (0.8–7.6)	37.6 % (20.5–58.4)
Incidence of TM Use (per year)			
1–5 times	47.2 % (38.8–55.7)	32.0 % (19.9–47.1)	45.3 % (27.8–64.0)
6–10 times	7.7 % (4.6–12.6)	17.0 % (7.8–33.0)	5.2 % (1.5–16.1)
>10 times	5.4 % (3.1–9.4)	7.1 % (2.6–18.0)	26.7 % (10.2–53.8)
Reasons for TM Use			
More Effective	83.3 % (74.6–89.4)	81.4 % (62.8–91.9)	79.6 % (40.4–95.7)
Lower Cost	60.1 % (48.2–70.9)	64.5 % (46.4–79.3)	55.2 % (32.2–76.2)
Easier to Access	69.9 % (61.0–77.6)	61.6 % (45.0–75.8)	59.1 % (30.7–82.5)
Safer	43.4 % (31.6–56.1)	39.1 % (25.8–54.2)	39.5 % (20.4–62.5)
More Traditional/Religious	30.4 % (23.4–38.4)	29.7 % (18.0–44.8)	42.0 % (24.1–62.3)
Modes of Healthcare Access			
Medical Doctors	97.3 % (93.4–98.9)	97.2 % (85.3–99.5)	90.5 % (58.6–98.5)
Family and Elders	50.7 % (38.0–63.4)	55.4 % (34.4–74.6)	69.0 % (42.8–86.9)
Traditional Healers	5.6 % (2.7–11.0)	9.9 % (3.0–28.2)	22.9 % (8.2–49.7)
Pharmacists	19.9 % (11.9–31.3)	22.0 % (10.5–40.5)	10.6 % (3.9–25.5)
Herbal Vendors	3.9 % (1.1–12.2)	11.4 % (5.7–21.5)	5.1 % (1.5–16.1)
Friends/Neighbors	17.5 % (10.4–27.8)	16.7 % (9.7–27.4)	5.2 % (1.7–15.3)
TM Use			
…for Symptomatic Ailments	57.3 % (49.2–65.0)	45.1 % (28.8–62.6)	79.1 % (60.8–90.2)
…for Chronic Diseases	27.7 % (19.1–38.0)	24.5 % (12.9–41.5)	57.0 % (29.4–80.9)
…for Reproductive/Fertility Ailments	22.8 % (14.6–33.9)	19.7 % (8.8–38.5)	49.5 % (28.2–70.9)
…for Malaria/Febrile Illnesses	56.7 % (43.1–69.4)	70.9 % (58.2–80.9)	59.2 % (28.7–84.0)
…for Spiritual/traditional uses	8.6 % (4.5–15.7)	7.0 % (2.2–20.2)	9.4 % (3.5–22.8)
…for Neurologic Illnesses	19.5 % (11.3–31.4)	19.6 % (10.2–34.5)	38.2 % (18.9–62.2)
…for Urogenital Conditions	15.7 % (9.8–24.4)	16.7 % (8.2–31.0)	26.0 % (10.6–51.1)
…for Cancers	14.7 % (6.6–29.7)	9.0 % (3.0–23.8)	22.9 % (8.0–50.6)
…for Disease Prevention	5.9 % (3.3–10.3)	0.7 % (0.1–5.8)	11.4 % (3.0–34.9)
…for Worms/Parasites	11.1 % (6.9–17.4)	15.9 % (9.3–25.9)	22.0 % (8.7–45.7)
Modes of TM Use			
Mix with water	83.0 % (77.4–87.4)	85.7 % (75.2–92.3)	79.8 % (54.6–92.9)
Drink as a tea	61.1 % (52.7–68.9)	47.5 % (31.0–64.5)	62.7 % (35.0–83.9)
Drink as a soup	46.1 % (36.6–55.8)	35.2 % (25.5–46.4)	65.5 % (38.3–85.3)
Chew from the plant	57.5 % (46.4–67.9)	47.3 % (31.9–63.3)	58.3 % (41.6–73.3)
Drink with milk	22.8 % (15.7–31.9)	15.7 % (7.5–30.0)	39.2 % (19.2–63.6)
Bath	26.2 % (17.7–37.0)	30.3 % (20.7–42.0)	45.0 % (19.3–73.8)
Inhalation	33.9 % (26.5–42.1)	39.2 % (26.8–53.1)	42.5 % (24.0–63.3)
Powders	17.4 % (9.7–29.2)	22.9 % (11.8–39.7)	25.3 % (9.9–51.0)
As foods to be eaten	2.4 % (0.8–6.8)	7.7 % (2.0–25.5)	4.2 % (1.1–14.9)
Pill/Vitamin form	0.6 % (0.2–1.7)	1.4 % (0.3–6.7)	2.4 % (0.4–13.0)
Lotions/Creams	5.3 % (2.9–9.5)	5.8 % (2.1–15.1)	21.0 % (5.9–53.2)

Chronic Diseases: Hypertension, Heart problems, Diabetes, or Body Swelling

Reproductive/Fertility Ailments: Sexual Arousal/Virility, Menstrual Problems, Pregnancy Termination, or Fertility/Impotence

Neurologic illnesses: Epilepsy, Mental Confusion, or Depression

Spiritual/Traditional: Peace of mind/Ward off curses, Protection from ‘evil eyes’, Unexplained Illnesses, or ‘To Improve Luck’

Symptomatic Ailments: Increase Strength, Constipation, Increase energy, Digestion/Stomach problems, Fatigue, Arthritis/joint pains, Flu/Cold symptoms, Headaches, or Skin problems

Urogenital: Kidney problems or Urinary problems

Adults with DM reported many reasons for the use of TMs (Table 2). When compared to biomedicines, the prevalence of TMs being reported as more effective was 79.6 % (95 % CI 40.4-95.7 %), as easier to access 59.1 % (95 % CI 30.7–82.5 %), as having lower costs 55.2 % (95 % CI 32.2–76.2 %), as being more traditional/religious 42.0 % (95 % CI 24.1–62.3 %), and as being safer was 39.5 % (95 % CI 20.4–62.5 %).

Community members with DM accessed healthcare from several sources (Table 2). Although most of them reported accessing healthcare from medical doctors (90.5 %; 95 % CI 58.6–98.5 %), many also reported accessing healthcare from family and elders, traditional healers, and pharmacists. Some also reported seeking healthcare from herbal vendors (5.1 %; 95 % CI 1.5–16.1 %) and friends/neighbors (5.2 %; 95 % CI 1.7–15.3 %). Individuals with DM reported using TMs most commonly for symptomatic ailments, malaria/febrile illness, chronic diseases, neurologic conditions, and reproductive/fertility ailments. The most common modes reported were mixing with water, tea, soup, or milk. Less frequently, they were reported as being inhaled, chewed, used with baths, lotions/creams, powders, or pill forms.

**Fig. 1:**
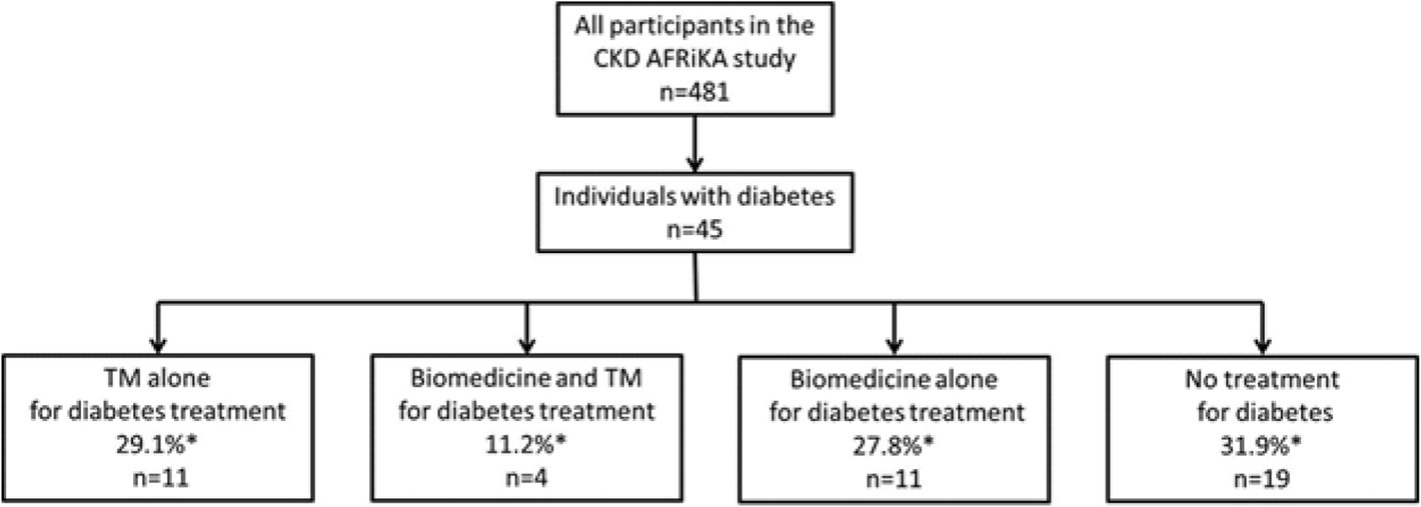
Treatment choices for diabetes care among individuals with diabetes in Northern Tanzania; CKD AFRiKA, 2014. *Prevalence rates were sample-balanced using age- and gender-weights based on the 2012 district-level census data

Many individuals were using TMs specifically for the treatment of their DM. The prevalence of TMs as a treatment choice for DM specifically was 40.3 % (95 % CI 20.5–63.9). The prevalence of TMs alone as the treatment choice for DM was 29.1 % (95 % CI 13.4–52.0) which was comparable to the prevalence of biomedicine alone (27.8 %; 95 % CI 13.0–49.8) as the treatment choice for DM (Fig. 1). Most commonly, individuals with DM were receiving no treatment (31.9 %; 95 % CI 14.4–56.5 %), either in the form of TM or biomedicine.

The prevalence of TM use was also high (56.7 %; 95 % CI 43.4–69.2 %) among adults at increased risk for DM (Table 2). Most reported using TMs 1–5 times per year (32.0 %; 95 % CI 19.8–47.2 %) or 6–10 times per year (17.0 %; 95 % CI 7.8–33.0 %). However, unlike adults with DM, those at risk for DM had a lower prevalence of biomedicine use (4.3 %; 95 % CI 1.7–10.4 %).

### Traditional medicines used for the treatment of diabetes in Northern Tanzania

We identified 168 plant-based TMs referenced by participants as part of the qualitative sessions and open-ended structured survey items. Three of these TMs were directly referenced as being used for the treatment of DM: Moringa (*Moringa oleifera*), lemongrass (*Cymbopogon citrullus*), and African redwood (*Hagenia abyssinica*) (Table 3 & Additional File 2). We further identified five plant-based TMs relevant in the treatment and management of DM. These were referenced as being in use by the local population for other conditions (Table 4 & Additional File 2): Aloe (*Aloe vera)*, Horsewood (*Clausena anisata*), Pigeon pea (*Cajanus cajan*), African wormwood (*Artimisia afra*), and Avocado (*Persea americana*). All of the TMs were identified as relevant for DM care had a wide-range of effects that could be potentially beneficial or harmful.

**Table 3:** Plant-based traditional medicines used for treatment of diabetes in the Kilimanjaro Region, Tanzania

Nomenclature			Uses in other African communities	Active Compounds and Pharmacology	Plant Parts in Use	Potential Side Effects and Toxicities
Scientific	English Common Name(s)	Local Vernacular				
Moringa oleifera^a^	Moringa; Drumstick tree	Mlonge	Senegal: stimulates breastmilk production, diabetes, anxiety, diarrhea and dysentery, colitis, gonorrhea, and various skin infections Chad: nutritional supplementation Nigeria and Benin: toothaches, GI ailments (dyspepsia, ulcers, and aiding digestion), poor vision, joint pains, diabetes, anemia, hypertension, paralysis, and helminthic infestation Uganda: diabetes, hypertension, HIV/AIDS-related symptoms, stimulates breastmilk production	Leaf extracts have glucose metabolism effects: modulates gene-expression of gluconeogenic liver enzymes, and regenerates pancreatic beta cells Nitrile and mustard oil glycosides: lowers blood pressure Seed kernels: bronchodilatory properties CNS effects: increases glutamate and serotonin; decreases norepinephrine and dopamine; anti-pyretic properties Anti-oxidative properties: may prevent drug-induced nephrotoxicity, myocardial damage, and gastric mucosal irritation Active compounds: salicylic and ferulic acids, flavonoids, phenolic acids, glucosinolates and isothiocanates, tannins and saponins	Flowers Pods/seeds Roots Leaves (Commonly grounded into powder for mixing)	-Abortifacient: causes uterine contractions -Inhibits CYP3A4 (inhibits metabolism of anti-diabetic drugs in the meglitinide class) -Chronic kidney disease (decline in glomerular filtration rate) -Hepatotoxicty (potential at high doses) -Paralysis
Cymbopogon Citrullus^a^	Lemongrass	Mchaichai	Southern Africa: diabetes, oral thrush, anti-tussive, anti-emetic, antiseptic, arthritis West Africa (Cameroon & Nigeria): antipyretic/anti-malarial, stimulant, anti-spasmodic, jaundice Mauritius: common cold, pneumonia, fever, GI ailments and dyspepsia	Oil extracts: anti-bacterial, anti-amebic, anti-fungal, antimalarial, anti-protozoal, and antifilarial effects Phenol and flavonoids: antioxidative Citral: insect repellent Active compounds: terpenes, alcohols, ketons, aldehyde, flavanoids, phenols, citral	Leaves Stem Oil extract	-Volume depletion -Diarrhea -Somnolence -Chronic kidney disease (decline in glomerular filtration rate) -Gastritis -Hepatotoxicty (potential) -Hypoglycemia
Hagenia abyssinica^a^	African redwood; East African rosewood	Enjani engashe (Maasai)	Ethiopia: Helminthic infections, Typhoid fever, wound healing, epilepsy, sexually transmitted diseases, and symptomatic ailments (dyspepsia, diarrhea, common cold, and cough)	Essential oils: trypanocidal (anti-spasmodic) and cytotoxic (in vitro activity against leukemic and adenocarcinoma cell lines) Active compounds: kosin (a phloroglucinol), & quercetin glucuronides	Flower and leaf extracts	-Hepatotoxicity -Diarrhea and volume depletion - Gastritis -Optic atrophy (blindness) -Abortifacient

*CNS* central nervous system, *CYP3A4* Cytochrome P450 3A4, *GI* gastrointestinal

^a^References are available in Additional file 2

**Table 4:** Plant-based traditional medicines relevant for the treatment and management of diabetes in Kilimanjaro Region, Tanzania

Nomenclature			Uses in other African communities	Active Compounds and Pharmacology	Plant Parts used	Potential Side Effects and Toxicities
Scientific	Common Name(s)	Local Vernacular				
Aloe vera (ferox and secundiflora species)^a^	Cape aloes, Aloe Vera	Aloe, Alovera	Southern Africa: arthritis, burns/skin conditions, hypertension, purging/laxative, dyspepsia, anti-inflammatory, cosmetics, eye ailments/conjunctivitis, sexually transmitted diseases, infertility, impotence East Africa (Kenya, Uganda, Ethiopia, and Tanzania): malaria, purging/laxative for cleansing purposes, dyspepsia, skin ulcerations/wound healing including burns, HIV/AIDS, cosmetic, infertility, anti-parasitic	Gel: Prostaglandin- and bradykinase-mediated anti-inflammatory activity. Aloin leaf extracts: increases GI motility and induces emesis Active compounds: glucomannans, thiamine, niacin, riboflavin, bradykinase, anthraquinone glycosides (aloin, barbaloin)	Gel extract Leaves Rind Stem	-Volume depletion and electrolyte imbalance -Hypoglycemia Hyperpigmentation and photosensitivity -Hepatotoxicity -Acute tubular necrosis -Acute interstitial nephritis
Clausena anisata^a^	Horsewood	Mjafari	West Africa: bacterial and fungal infections of the skin including boils, ringworm, and eczema East Africa (Tanzania): oral candidiasis, fungal infections of the skin, and epilepsy Southern Africa: epilepsy, arthritis, rheumatism and other inflammatory conditions, hypertension, heart failure and other heart ailments, schistosomiasis, taeniasis and other parasitic infections, constipation and dyspepsia; malaria and other febrile conditions, headaches, eye ailments/conjunctivitis, impotence and infertility	Leaf extracts inhibit ACE: may lower blood pressure Bacteriostatic against gram positive and gram negative bacteria; Fungicidal activity against Aspergilus fumigatus Antiplasmodial: in-vitro dose-dependent schizonticidal effect of leaf extracts on parasitemia In vitro activity against leukemic cell lines Anti-HIV1/2 effects: dose-dependent inhibition of reverse transcriptase and taq polymerase enzymes Hypoglycemic properties (reduction in basal blood glucose levels); Anti-convulsant; ACE inhibition; Cyclooxygenase-1 and -2 (weak) inhibition Active compounds: Clausamine, carbazole alkaloids (girinimbine, murrayamine-A, and ekeberginine), flavonoids, monoterpenes, and coumarins	Leaf, stem, and root extracts	-Heavy metal bio-accumulation (Iron, cadmium, manganese) -Hypoglycemia -Gastritis
Cajanus cajan^a^	Pigeon pea	Majani ya mbaazi	Ghana: diabetes, dysentery, hepatitis, measles, dysmenorrhea Nigeria: wound healing, aphthae, bedsores, and malaria/fever	Antibacterial activity; hypocholesterolemic effects (diet-induced); inhibits CNS voltage-gated Na channels; induces apoptosis in human breast cancer cells via a ROS-mediated mitochondrial pathway; inhibits TNF-α and IL-1β production Glycemic profile: leaves induce hyperglycemia, seeds induce hypoglycemia Active compounds: Cajanuslactone, stilbene, pinostrobin, cajanol	Leaves Seeds	-CNS depression -Somnolence -Heavy metal bio-accumulation (arsenic, copper, aluminum) -Bronchospasm -Hypoglycemia
Persea Americana^a^	Avocado	Mparachichi, Mwembe, Mafuta	West Africa (Nigeria, Togo, Ivory Coast): anti-diarrheal, diabetes/hyperglycemia, anti-inflammatory, wound healing, antiepileptic, exhaustion, hypertension, gastritis/dyspepsia East Africa (Kenya, Uganda, Tanzania, Zimbabwe, Mozambique): dengue vector control, diarrhea, sore throat, menstrual regulation, hair growth, epilepsy, toothaches, wound healing, tuberculosis, neuralgia)	Anti-inflammatory, limiting lowering (β-Carotene and fatty acids), anticonvulsive (via gabanergic effects) & vasodilatory properties; Inhibits alpha-amylase and enhance glycogenesis; acetogenins inhibit platelet aggregation; larvicidal to Aedes aegypti Active Compounds: Tannins, saporins, alkanols (aliphatic acetogenins), terpenoids, coumarin	Leaves Fruits Seeds Rind Bark	-Increased risk of bleeding when combined with other anti-coagulants -Hypoglycemia -Hyperkalemia (especially among those with impaired kidney function)
Artemisia afra^a^	African wormwood	Fivi, Majani mapana artemisia	Southern Africa: coughs, colds, sore throat, gastritis/reflux, hemorrhoids, fevers, malaria, asthma, diabetes	Lowers blood glucose, improves glucose tolerance and balance in lipid metabolism; anti-oxidative properties bactericidal against gram positive and gram negative bacteria	Roots stems leaves	- Chronic kidney disease (decline in glomerular filtration rate) -Acute tubular necrosis -Hypoglycemia

*GI* gastro-intestinal, *ACE* angiotensin converting enzyme, *TNF* tumor necrosis factor, *IL* interleukin, *CNS* central nervous system, *ROS* reactive oxygen species

^**a**^References are available in Additional file 2

## Discussion

The prevalence of TM use was high among individuals with DM and those at increased risk for DM in Northern Tanzania, and the plant-based TMs commonly used towards DM care had numerous effects. Individuals with and at risk for DM sought healthcare advice from many non-biomedical practitioners including family members, community and tribal elders, friends, traditional healers, and herbal vendors. In addition, TMs were a common treatment choice for DM, and many individuals with DM used TM and biomedicine concurrently.

TMs are now recognized globally as important components of healthcare systems [9–11, 13]. In sub-Saharan Africa, TMs remain critical in meeting the healthcare needs for many individuals. However, with the rising burden of NCDs across sub-Saharan Africa [3, 4], the continued role and importance of TMs in NCD management is less clear. As such, our study, which highlights TM practices among individuals with diabetes in northern Tanzania, fills an important gap. In resource-limited health facilities across Tanzania and sub-Saharan Africa, taking a patient- or community-centered approach that is sensitive to treatment preferences may be most effective, especially for improving NCD management. For example, understanding high and low risk plant-based TMs may be one approach to incorporate TM preferences into an NCD management program [22–24]. Additionally, educating TM practitioners and other key stakeholders about safe and appropriate NCD care, including timely referrals to biomedical practitioners, may also be an effective approach to incorporate TM preferences into NCD management programs. Other potential strategies could include training community health workers to deliver locally-relevant medical care to patients with DM, developing community-based outreach programs that include DM education consistent with local health belief systems and practices, or incorporating family members and caregivers into health interventions [4, 14].

In order to develop such programs and policies, we need to facilitate healthcare decisions that can leverage the benefits of TMs while mitigating the risks. As such, a better understanding of the clinical safety, efficacy, and quality of TMs in local settings is urgently needed. Moreover, governments should establish a framework for accreditation, registration, and regulation of TM practitioners [22, 23]. Among the TMs that we identified, they all had numerous pharmacologically active compounds and a variety of effects. Potential beneficial effects for individuals with DM included blood pressure lowering, anti-microbial, anti-inflammatory, anti-oxidative, and lipid lowering properties. Additionaly, several of them, including *Cymbopogon cirtullus* [25, 26]*, Moringa oleifera* [27]*, Aloe vera* [28]*, Clausean anista* [29]*, Cajanus cajan* [30, 31]*, Persea americana* [32] and *Artimisia afra* [33], also had direct glucose-lowering effects, with some having specific effects on glucose metabolism.

On the other hand, all the TMs we identified also had potentially harmful side effects, including nephrotoxicy, optic atrophy, hepatotoxicity, and volume depletion among others, which may be particularly relevant for individuals with DM who often suffer several co-morbidities. Our findings extend the work of others across the region by stressing the importance of equipping both biomedical providers and traditional healers with resources and education that can facilitate evidence-based discussions regarding TM use among their patient populations. For example, *Clausena anisata* (Horsewood) has numerous potential benefits for individuals with DM, including blood pressure lowering through inhibition of angiotensin-converting enzyme, which may be particularly important among those with proteinuria [34]. However, it also exhibits a dose-dependent inhibition of many reverse transcriptase inhibitors commonly used in the treatment of HIV [35]. Also, *Aloe vera* is commonly and effectively used topically to treat burns and minor skin conditions, but when boiled and ingested in large amounts to treat dyspepsia, it can cause acute kidney and liver injuries. Therefore, through understanding these important effects and practices, providers caring for individuals with DM may be able to tailor interventions in ways that are effective yet also patient-centered and sensitive to healthcare-seeking preferences.

We noted a few limitations in our study. Our cross-sectional design does not allow causal pathways to be established and associations may be confounded by unmeasured variables. Additionally, non-response bias may be present, and to reduce this potential bias we used sample-balanced weights when reporting prevalence estimates. Also, around culturally sensitive topics such as TMs, reporting and recall bias are a concern. To reduce these biases, we used only local native surveyors who spoke Swahili as their first language, conducted the interviews in private when possible, and pre-tested the survey instruments for content validity and design flaws. Misclassification of disease may also be present, and though we expect most misclassification to be non-differential, the measurement we used to diagnose diabetes (HbA1c) has not been validated in this population. As such, the sensitivity and specificity of the test at a cutoff value of 7.0 % (53 mmol/mol) are not known for this population.

## Conclusion

In conclusion, the prevalence of TM use was high among individuals with DM as well as those at increased risk for DM in northern Tanzania, and many of these individuals use TMs and biomedicines together. Individuals with DM sought healthcare advice from several sources, and biomedical providers need to be aware of these practices, especially as they pertain to TM use. The TMs commonly used by individuals with DM in northern Tanzania have a wide range of effects, and understanding these effects will help shape biomedical practitices and public health policies that are effective yet also patient-centered and sensitive to local TM preferences.

## Abbreviations

CI, confidence intervals; CKD AFRiKA, Comprehensive Kidney Disease Assessment for Risk Factors, epidemiology, Knowledge, and Attitudes; COPD, chronic obstructive pulmonary disease; DM, diabetes mellitus; FGD, focus group discussion; HbA1c, Hemoglobin (Hb) A1c; HIV, human immunodeficiency virus; KCMC, Kilimanjaro Christian Medical College; NCD, non-communicable disease; TM, traditional medicine

## Additional files

Additional file 1: Structured Survey Instrument for the use of Traditional Medicines (English and Swahili). (DOCX 454 kb) Additional file 2: Supplementary references for Traditional Medicines used for the treatment of diabetes in Northern Tanzania. (DOCX 28 kb)
