# Supplementary material for: Traditional medicine practices among community members with diabetes mellitus in Northern Tanzania: an ethnomedical survey
Source: BMC Complement Altern Med. 2016 Aug 11;16:282. doi: 10.1186/s12906-016-1262-2 (PMC4982437; doi:10.1186/s12906-016-1262-2)
Supplement: Additional file 2: — Supplementary references for Traditional Medicines used for the treatment of diabetes in Northern Tanzania. (DOCX 28 kb) [file 12906_2016_1262_MOESM2_ESM.docx]

**Traditional Medicine Practices Among Community Members with Diabetes Mellitus in Northern Tanzania: An Ethnomedical Survey**

**Additional file 1:**

**Supplementary references for Traditional Medicines used for the treatment of diabetes in Northern Tanzania**

Joseph Lunyera MBChB, MSc^1^; Daphne Wang, BS^1^; Venance Maro, MD, MMed^2^; Francis Karia, MBA, MSc^2^; David Boyd, PhD^1^; Justin Omolo, PhD^3^; Uptal D Patel, MD^1,4,5^; and John W Stanifer, MD, MSc^1,4,5^ for the Comprehensive Kidney Disease Assessment For Risk factors, epidemiology, Knowledge, and Attitudes (CKD AFRiKA) Study

1. Duke Global Health Institute, Duke University; Durham, NC United States
2. Kilimanjaro Christian Medical University College; Moshi, Tanzania
3. National Institute for Medical Research, Dar es Salaam, Tanzania
4. Division of Nephrology, Department of Medicine, Duke University School of Medicine; Durham, NC United States
5. Duke Clinical Research Institute, Duke University; Durham, NC United States

Correspondence:

Joseph Lunyera

Duke Global Health Institute

310 Trent Drive,

Durham, NC 27705

***Moringa oleifera***

1. Kasolo JN, Bimenya GS, Ojok L, Ochieng J, Ogwal-Okeng JW. Phytochemicals and uses of Moringa oleifera leaves in Ugandan rural communities. *J. Med. Plant Res.* 2010, 4, 753–757.
2. Leone A, Fiorillo G, Criscuoli F, Ravasenghi S, Santagostini L, Fico G, Spadafranca A, Battezzati A, Schiraldi A, Pozzi F, di Lello S, Filippini S, Bertoli S. Nutritional Characterization and Phenolic Profiling of Moringa oleifera Leaves Grown in Chad, Sahrawi Refugee Camps, and Haiti. *Int J Mol Sci* 16: 18923-37, 2015.
3. Anwar F, Latif S, Ashraf M, Gilani AH. 2007. Moringa oleifera: a food plant with multiple medicinal uses. *Phytother Res* 21: 17-25, 2007.
4. Popoola JO, Obembe OO. Local knowledge, use pattern and geographical distribution of Moringa oleifera Lam. (Moringaceae) in Nigeria. *J Ethnopharmacol* 150: 682-91, 2013.
5. Abd El Latif A, El Bialy Bel S, Mahboub HD, Abd Eldaim MA. Moringa oleifera leaf extract ameliorates alloxan-induced diabetes in rats by regeneration of β cells and reduction of pyruvate carboxylase expression. *Biochem Cell Biol*. 2014 Oct; 92(5): 413-9.
6. Leone A, Spada A, Battezzati A, Schiraldi A, Aristil J, Bertoli S. Cultivation, Genetic, Ethnopharmacology, Phytochemistry and Pharmacology of Moringa oleifera Leaves: An Overview. *Int J Mol Sci* 16: 12791-835, 2015.
7. Duthie GG, Wood AD. Natural salicylates: foods, functions and disease prevention. *Food Funct* 2: 515-20, 2011.
8. Adeyemi OS, Elebiyo TC. Moringa oleifera Supplemented Diets Prevented Nickel-Induced Nephrotoxicity in Wistar Rats. *J Nutr Metab*. 2014; 2014: 958621.

***Cymbopogon citrullus***

1. Ekpenyong CE, Daniel NE, Antai AB. Effect of lemongrass tea consumption on estimated glomerular filtration rate and creatinine clearance rate. *J Ren Nutr*. 2015; 25(1): 57–66.
2. Carlini EA, Contar JP, Silva-Filho AR, da Silveira-Filho NG, Frochtengarten ML, Bueno OF. Pharmacology of lemongrass (Cymbopogon citratus Stapf): effects of teas prepared from the leaves on laboratory animals. *J Ethnopharmacol*. 1986; 17(1): 37–64.
3. Shah G, Shri R, Panchal V, Sharma N, Singh B, Mann AS. Scientific basis for the therapeutic use of Cymbopogon citratus, stapf (Lemon grass). *J Adv Pharm* Technol Res. 2011; 2(1): 3–8.
4. Maia MF, Moore SJ. Plant-based insect repellents: a review of their efficacy, development and testing. *Malar J*. 2011; 10: S11.
5. Boaduo NK, Katerere D, Eloff JN, Naidoo V. Evaluation of six plant species used traditionally in the treatment and control of diabetes mellitus in South Africa using in vitro methods. *Pharm Biol*. 2014; 52(6): 756–61.
6. Dike IP, Obembe OO, Adebiyi FE. Ethnobotanical survey for potential anti-malarial plants in south-western Nigeria. *J Ethnopharmacol*. 2012; 144(3): 618–26.
7. Fandohan P, Gnonlonfin B, Laleye A, Gbenou JD, Darboux R, Moudachirou M. Toxicity and gastric tolerance of essential oils from Cymbopogon citratus, Ocimum gratissimum and Ocimum basilicum in Wistar rats. *Food Chem Toxicol*. 2008; 46(7): 2493–7.
8. Adeneye AA, Agbaje EO. Hypoglycemic and hypolipidemic effects of fresh leaf aqueous extract of Cymbopogon citratus Stapf. in rats. *J Ethnopharmacol*. 2007; 112(3): 440–4.
9. Gold CH. Acute renal failure from herbal and patent remedies in Blacks. *Clin Nephrol*. 1980; 14(3): 128–34.

***Hagenia abyssinica***

1. Assefa B, Glatzel G, Buchmann C. Ethnomedicinal uses of Hagenia abyssinica (Bruce) J.F. Gmel. among rural communities of Ethiopia. *J Ethnobiol Ethnomed* 2010, 6: 20.
2. Nibret E, WinkM. Trypanocidal and antileukaemic effects of the essential oils of Hagenia abyssinica, Leonotis ocymifolia, Moringa stenopetala, and their main individual constituents. *Phytomedicine* 2010, 17: 911-20.
3. Woldemariam TZ, Fell AF, Linley PA, Bibby MC, Phillips RM. Evaluation of the anti-tumour action and acute toxicity of kosins from Hagenia abyssinica. *J Pharm Biomed Anal* 1992, 10: 555-60.
4. Arragie M, Metzner J, Bekemeier H. Antispasmodic effect of Hagenia abyssinica. *Planta Med* 1983, 47: 240-241.
5. Low G, Rogers LJ, Brumley SP, Ehrlich D. Visual deficits and retinotoxicity caused by the naturally occurring anthelmintics, Embelia ribes and Hagenia abyssinica. *Toxicol Appl Pharmacol* 1985, 81: 220-30.

***Aloe vera* (*ferox* and *secundiflora* species)**

1. Luyckx VA, Ballantine R, Claeys M, Cuyckens F, Van den Heuvel H, Cimanga RK, et al. Herbal remedy-associated acute renal failure secondary to Cape aloes. *Am J Kidney Dis*. 2002; 39(3), e13.
2. Aloes in Kenya. International Workshop on the Convention of International Trade in Endangered Species of Wild Fauna and Flora; Cancun, Mexico 2008.
3. Eshun K, He Q. Aloe vera: a valuable ingredient for the food, pharmaceutical and cosmetic industries–a review. *Crit Rev Food Sci Nutr*. 2004; 44(2): 91–6.
4. Mahomoodally MF. Traditional medicines in Africa: an appraisal of ten potent african medicinal plants. *Evid Based Complement Alternat Med*. 2013; 2013: 617459.
5. Chen W, Van Wyk B-E, Vermaak I, Viljoen AM. Cape aloes—a review of the phytochemistry, pharmacology and commercialisation of aloe ferox. *Phytochem Lett*. 2012; 5(1): 1–12.
6. Nanyingi MO, Mbaria JM, Lanyasunya AL, Wagate CG, Koros KB, Kaburia HF, et al. Ethnopharmacological survey of Samburu district, Kenya. *J Ethnobiol Ethnomed*. 2008; 4: 14.
7. Vogler BK, Ernst E. Aloe vera: a systematic review of its clinical effectiveness. *Br J Gen Pract*. 1999; 49(447): 823–8.

***Clausena anisata***

1. Ojewole JA. Hypoglycaemic effect of Clausena anisata (Willd) Hook methanolic root extract in rats*. J Ethnopharmacol* 2002, 81: 231-237.
2. Moshi MJ, Kagashe GA, Mbwambo ZH. Plants used to treat epilepsy by Tanzanian traditional healers. *J Ethnopharmacol*. 2005 Feb 28; 97(2): 327-36.
3. Duncan AC, Jäger AK, van Staden J. Screening of Zulu medicinal plants for angiotensin converting enzyme (ACE) inhibitors. *J Ethnopharmacol*. 1999 Dec 15; 68(1-3): 63-70.
4. Adesina SK. The isolation and identification of anticonvulsant agents from Clausenia anisata and Afraegle paniculata. *Fitoterapia* 1982 (0367-326X), 53 (3), p.63.
5. Okokon JE, Etebong EO, Udobang JA, Essien GE. 2012. Antiplasmodial and analgesic activities of Clausena anisata. *Asian Pac J Trop Med* 2012, 5: 214-219.
6. Ito C, Itoigawa M, Aizawa K, Yoshida K, Ruangrungsi N, Furukawa H. Gamma-lactone carbazoles from Clausena anisata. *J Nat Prod* 2009, 72: 1202-1204.
7. Agyepong N, Agyare C, Adarkwa-Yiadom M, Gbedema SY. Phytochemical investigation and anti-microbial activity of clausena anisata (willd), hook. *Afr J Tradit Complement Altern Med* 2014, 11: 200-209.
8. Ayisi NK, Nyadedzor C. Comparative in vitro effects of AZT and extracts of Ocimum gratissimum, Ficus polita, Clausena anisata, Alchornea cordifolia, and Elaeophorbia drupifera against HIV-1 and HIV-2 infections. *Antiviral Res*. 2003 Mar; 58(1): 25-33.

***Cajanus cajan***

1. Jaiswal D, Rai PK, Kumar A, Watal G. Study of glycemic profile of Cajanus cajan leaves in experimental rats. *Indian J Clin Biochem* 23: 167-70, 2008.
2. Annan K, Kojo AI, Cindy A, Samuel AN, Tunkumgnen BM. Profile of heavy metals in some medicinal plants from Ghana commonly used as components of herbal formulations. *Pharmacognosy Res* 2010, 2: 41-44.
3. Patel NK, Bhutani KK. Pinostrobin and Cajanus lactone isolated from Cajanus cajan (L.) leaves inhibits TNF-alpha and IL-1beta production: in vitro and in vivo experimentation. *Phytomedicine* 2014, 21: 946-53.
4. Misra A, Kumar R, Mishra V, Chaudhari BP, Tripathi A, Das M, Dwivedi PD. Partial characterization of red gram (Cajanus cajan L. Millsp) polypeptides recognized by patients exhibiting rhinitis and bronchial asthma. *Food Chem Toxicol* 2010, 48: 2725-36.
5. Ezike AC, Akah PA, Okoli CC, Okpala CB. Experimental evidence for the antidiabetic activity of cajanus cajan leaves in rats. *J Basic Clin Pharm* 2010, 1: 81-84.
6. Luo QF, Sun L, Si JY, Chen DH. Hypocholesterolemic effect of stilbenes containing extract-fraction from Cajanus cajan L. on diet-induced hypercholesterolemia in mice. *Phytomedicine* 2008, 15: 932-939.
7. Luo M, Liu X, Zu Y, Fu Y, Zhang S, Yao L, Efferth T. Cajanol, a novel anticancer agent from Pigeonpea [Cajanus cajan (L.) Millsp.] roots, induces apoptosis in human breast cancer cells through a ROS-mediated mitochondrial pathway. *Chem Biol Interact* 2010, 188: 151-60.

***Persea Americana***

1. Musabayane CT. The effects of medicinal plants on renal function and blood pressure in diabetes mellitus. *Cardiovasc J Afr*. 2012; 23(8): 462–8.
2. Ojewole JA, Kamadyaapa DR, Gondwe MM, Moodley K, Musabayane CT. Cardiovascular effects of Persea americana Mill (Lauraceae) (avocado) aqueous leaf extract in experimental animals. *Cardiovasc J Afr*. 2007; 18(2): 69–76.
3. Yasir M, Das S, Kharya MD. The phytochemical and pharmacological profile of Persea americana Mill. *Pharmacogn Rev*. 2010; 4(7): 77–84.
4. Rodriguez-Sanchez DG, Flores-Garcia M, Silva-Platas C, Rizzo S, Torre-Amione G, De la Pena-Diaz A, et al. Isolation and chemical identification of lipid derivatives from avocado (Persea americana) pulp with antiplatelet and antithrombotic activities. *Food Funct*. 2015; 6(1): 193–203.
5. Ojewole JA, Amabeoku GJ. Anticonvulsant effect of Persea americana Mill (Lauraceae) (Avocado) leaf aqueous extract in mice. *Phytother Res*. 2006; 20(8): 696–700.
6. Adeyemi OO, Okpo SO, Ogunti OO. Analgesic and anti-inflammatory effects of the aqueous extract of leaves of Persea americana mill (lauraceae). *Fitoterapia*. 2002; 73(5): 375–80.
7. Karou SD, Tchacondo T, Djikpo Tchibozo MA, Abdoul-Rahaman S, Anani K, Koudouvo K, et al. Ethnobotanical study of medicinal plants used in the management of diabetes mellitus and hypertension in the Central Region of Togo. *Pharm Biol*. 2011; 49(12): 1286–97.
8. Tabuti JR, Kukunda CB, Waako PJ. Medicinal plants used by traditional medicine practitioners in the treatment of tuberculosis and related ailments in Uganda. *J Ethnopharmacol*. 2010; 127(1): 130–6.
9. Lim T. Edible Medicinal and Non-Medical Plants. New York: *Springer*; 2012.

***Artemisia afra***

1. Sunmonu TO, Afolayan AJ. Evaluation of Antidiabetic Activity and Associated Toxicity of Artemisia afra Aqueous Extract in Wistar Rats. *Evid Based Complement Alternat Med* 2013: 929074, 2013.
2. Sunmonu, T. O. & Afolayan, A. J. 2010. Protective effect of Artemisia afra Jacq. on isoproterenol-induced myocardial injury in Wistar rats. *Food Chem Toxicol* 48: 1969-72.
3. Mukinda JT, Syce J. Acute and chronic toxicity of the aqueous extract of Artemisia afra in rodents. *J Ethnopharmacol* 2007, 112: 138-44.
4. Noori A, Amjad L, Yazdani F. The effects of Artemisia deserti ethanolic extract on pathology and function of rat kidney. *Avicenna J Phytomed* 2014, 4: 371-376.
